# Analysis of *Dipylidium caninum* tapeworms from dogs and cats, or their respective fleas: Part 1. Molecular characterization of *Dipylidium caninum*: genetic analysis supporting two distinct species adapted to dogs and cats

**DOI:** 10.1051/parasite/2018028

**Published:** 2018-05-28

**Authors:** Michel Labuschagne, Frédéric Beugnet, Steffen Rehbein, Jacques Guillot, Josephus Fourie, Dionne Crafford

**Affiliations:** 1 Boehringer Ingelheim Animal Health, 29 Av Tony Garnier, 69007 Lyon France; 2 Clinomics, P.O. Box 11186, Universitas, 9321, Bloemfontein South Africa; 3 Boehringer Ingelheim Animal Health, Kathrinenhof Research Centre, Walchenseestr. 8-12, 83101 Rohrdorf Germany; 4 École Nationale Vétérinaire de Maisons-Alfort, 94704 Maisons-Alfort Cedex France; 5 Clinvet, P.O. Box 11186, Universitas, 9321, Bloemfontein South Africa

**Keywords:** *Dipylidium caninum*, mitochondrial genome, *Ctenocephalides felis*, dogs, cats, genotypes

## Abstract

A 28S rDNA PCR detection assay was previously developed to identify *Dipylidium caninum* DNA inside single fleas collected from both cats and dogs. Sequence analysis of the 28S rDNA fragment indicated two genetically distinct variations of the target region. The two genotypes, so-called “*D.* *caninum* canine genotype” and “*D.* *caninum* feline genotype”, based on host origin, are further investigated and described in this paper. Restriction fragment length polymorphism (RFLP) analysis and hydrolysis probe-based genotyping assays were developed and validated for genotyping *D.* *caninum* DNA. The complete mitochondrial (mt) genome of the “feline genotype” was sequenced and compared to the *D.* *caninum* mt genome available in GenBank. The molecular characterization of *D.* *caninum* isolates collected from infected fleas, and also proglottids collected from dogs and cats, confirmed the existence of two distinct genotypes. These genotypes are related to host origin (dogs or cats), irrespective of their geographical origin, and they present a biological adaptation to their respective host, as confirmed by the comparison of biological development and host preference in another study. The genetic differences (Part 1, present paper) and biological observations (Part 2, in this journal) enabled us to suggest the existence of two distinct species within *D.* *caninum*, which will have to be clarified.

## Abstract (fr)

Un test PCR de détection de fragment de l’ADN ribosomal de *Dipylidium caninum* dans des puces récoltées sur chiens et chats a été précédemment développé. L’analyse des séquences de l’ADNr 28S a démontré la présence de deux génotypes distincts. Ces deux génotypes, appelés génotype canin et génotype félin sur la base de l’origine des isolats, sont étudiés et présentés dans cet article. L’hydrolyse de fragments d’ADN et l’analyse du polymorphisme de taille des sites de restriction ont été mise au point et validées pour génotyper l’ADN de *D.* *caninum*. Le séquençage complet du génome mitochondrial du génotype félin a été réalisé et comparé au génotype canin, dont le génome mitochondrial est disponible dans GenBank. L’analyse moléculaire des isolats de *D.* *caninum* collectés à partir de puces infectées, ou de proglottis issus de chiens et chats infestés confirme l’existence de deux génotypes distincts. Ces génotypes sont liés à l’hôte d’origine, chien ou chat, quelle que soit leur origine géographique, et ils présentent une adaptation biologique à leur hôte d’origine, comme confirmé dans une autre étude. Les différences génétiques (Partie 1, cet article) et les observations biologiques (Partie 2, dans ce journal) permettent de suggérer l’existence de deux espèces au sein de *D.* *caninum*, ce qui devra être clarifié.

## Introduction

*Dipylidium caninum* (Linnaeus, 1758), a globally distributed cestode, infects domestic cats and dogs [8], wild canids and felids [7,10], and occasionally humans [15]. In 1758, Linnaeus recognized the parasite and named it *Taenia canina*. In 1863, Leuckart erected the genus *Dipylidium*, but it was not until 1893 that the internal anatomy and observations of the life history of *Dipylidium caninum* was described by Diamare (in [16]). The cat flea, *Ctenocephalides felis*, is considered the main intermediate host of *D.* *caninum* [5]. Furthermore, *C.* *felis* has the ability to infest both dogs and cats. The dog flea (*Ctenocephalides canis*) also acts as an intermediate host, but exceptionally infests cats. The life cycle of *D.* *caninum* can be summarized as follows: flea larvae ingest eggs of *D.* *caninum*, followed by development of the egg to the metacestode stage inside the flea. When a canine or feline host ingests adult fleas infected with suitably developed metacestodes, the parasite establishes in the small intestine of its definitive host.

Several species belonging to the genus *Dipylidium* have historically been suggested based on morphological observations [16]. However, significant overlap in morphological traits led to recognition of a single species, namely *D.* *caninum* [16,18]. As a result, the genus *Dypilidium* is currently considered monotypic. However, in the absence of distinguishing morphological characters or significant overlap thereof, modern molecular techniques often allow us to differentiate hidden genetic lineages and cryptic species [e.g. 1,6,12,17,19]. A molecular approach would potentially be highly beneficial in investigating and confirming species status within the genus *Dipylidium*.

In 2014, Beugnet et al. [3] investigated the prevalence of *D.* *caninum* in fleas from client-owned cats and dogs in Europe, using a new PCR detection assay targeting a region within the 28S rDNA. The results confirmed the spread of *D.* *caninum sensu lato* in fleas of dogs and cats throughout Europe. Preliminary analyses indicated genetic differences between *D.* *caninum* metacestodes in fleas collected from dogs and cats, respectively. These preliminary analyses are described in the present article as well as all further molecular assessments that were performed in order to confirm the existence of multiple *D.* *caninum* genotypes.

Original sequence analysis of PCR products from *Dipylidium* infected fleas collected in 2011 and 2012 (Beugnet et al., 2014 [3]) indicated two 28S rDNA sequence variants of the target region. This preliminary analysis suggested that a host-specific preference may be applicable, and hence the two distinct 28S rDNA sequence variants were defined as the so-called “*D. caninum* canine genotype” and the “*D. caninum* feline genotype” [previously unpublished, see Tables 1–Tables 1 to 3]. The *D. caninum* canine genotype was found in > 95% cases in infected *C.* *felis* fleas collected on dogs and 100% of *C.* *canis* infected fleas, whereas the *D.* *caninum* feline genotype was identified in > 95% of *C.* *felis* infected fleas collected on cats. It was thus decided to further investigate these differences, with specific reference to possible genotype-host associations.

**Table 1:** Details on two distinct 28S rDNA sequence variants (“canine” and “feline”) defined from fleas infected by *Dipylidium caninum*, obtained from PCR products collected by Beugnet et al. [3] from Europe and South Africa.

Sample ID	Source	Geographic origin	Host	RFLP Genotype
CVML12/008/006/024	*C.* *felis*	Czech Republic	Cat	Canine
CVML12/008/023/002	*C.* *felis*	Slovenia	Cat	Feline
CVML12/008/023/003	*C.* *felis*	Slovenia	Cat	Feline
CVML12/008/023/004	*C.* *felis*	Slovenia	Cat	Feline
CVML12/008/023/005	*C.* *felis*	Slovenia	Cat	Feline
CVML12/008/023/006	*C.* *felis*	Slovenia	Cat	Feline
CVML12/008/023/009	*C.* *felis*	Slovenia	Cat	Feline
CVML12/008/023/010	*C.* *felis*	Slovenia	Cat	Feline
CVML12/008/034/003	*C.* *felis*	Slovenia	Cat	Feline
CVML12/008/041/001	*C.* *felis*	Slovenia	Cat	Feline
CVML12/008/060/007	*C.* *felis*	Portugal	Cat	Not determined
CVML12/008/105/002	*C.* *felis*	France	Cat	Canine
CVML12/008/118/002	*C.* *felis*	France	Cat	Not determined
CVML12/008/193/001	*C.* *felis*	France	Dog	Feline
CVML12/008/198/006	*C.* *felis*	France	Dog	Canine
CVML12/008/198/007	*C.* *felis*	France	Dog	Canine
CVML12/008/231/007	*C.* *felis*	France	Dog	Canine
CVML12/008/231/008	*C.* *felis*	France	Dog	Canine
CVML12/008/246/002	*C.* *felis*	France	Dog	Canine
CVML12/008/248/025	*C.* *felis*	France	Dog	Canine
CVML12/008/248/036	*C.* *felis*	France	Dog	Canine
CVML12/008/265/001	*C.* *felis*	Hungary	Cat	Feline
CVML12/008/265/010	*C.* *felis*	Hungary	Cat	Feline
CVML12/008/277/006	*C.* *felis*	Germany	Cat	Feline
CVML12/008/277/013	*C.* *felis*	Germany	Cat	Feline
CVML12/008/279/003	*C.* *felis*	Portugal	Cat	Feline
CVML12/008/279/014	*C.* *felis*	Portugal	Cat	Feline
CVML12/008/364/001	*C.* *felis*	Sicily	Dog	Canine
CVML12/008/365/004	*C.* *felis*	Sicily	Dog	Canine
CVML12/008/366/001	*C.* *felis*	Sicily	Dog	Canine
CVML12/008/373/002	*C.* *felis*	Sicily	Dog	Canine
CVML12/008/397/004	*C.* *felis*	Sicily	Dog	Canine
CVML12/008/474/013	*C.* *felis*	France	Dog	Canine
CVML12/008/523/005	*C.* *felis*	Sicily	Dog	Canine
CVML12/008/530/003	*C.* *felis*	Sicily	Dog	Canine
CVML12/008/539/002	*C.* *felis*	Sicily	Dog	Canine
CVML12/008/550/001	*C.* *felis*	Sicily	Dog	Canine
CVML12/008/598/002	*C.* *felis*	Sicily	Dog	Canine
CVML12/008/601/003	*C.* *felis*	Sicily	Dog	Canine
CVML12/008/601/004	*C.* *felis*	Sicily	Dog	Canine
CVML12/008/603/003	*C.* *felis*	Sicily	Dog	Canine
CVML12/008/632/002	*C.* *felis*	Sicily	Dog	Canine
CVML12/008/642/004	*C.* *felis*	Sicily	Dog	Canine
CVML12/008/651/002	*C.* *felis*	Sicily	Dog	Canine
CVML12/008/651/003	*C.* *felis*	Sicily	Dog	Canine
CVML12/008/653/002	*C.* *felis*	Sicily	Dog	Canine
CVML12/008/654/004	*C.* *felis*	Sicily	Dog	Canine
CVML12/008/664/005	*C.* *felis*	Sicily	Dog	Canine
CVML12/008/671/001	*C.* *felis*	France	Cat	Feline
CVML12/008/739/005	*C.* *felis*	Romania	Cat	Feline
CVML12/008/739/007	*C.* *felis*	Romania	Cat	Feline
CVML12/008/739/009	*C.* *felis*	Romania	Cat	Feline
CVML12/008/754/002	*C.* *felis*	Romania	Cat	Feline
CVML12/008/757/002	*C.* *felis*	Romania	Cat	Not determined
CVML12/008/758/002	*C.* *felis*	Romania	Cat	Feline
CVML12/008/758/003	*C.* *felis*	Romania	Cat	Feline
CVML12/008/758/004	*C.* *felis*	Romania	Cat	Feline
CVML12/008/791/001	*C.* *felis*	Hungary	Cat	Feline
CVML12/008/800/002	*C.* *felis*	Hungary	Cat	Feline
CVML12/008/800/009	*C.* *felis*	Hungary	Cat	Feline
CVML12/008/800/011	*C.* *felis*	Hungary	Cat	Feline
CVML12/008/803/001	*C.* *felis*	Hungary	Cat	Feline
CVML12/008/807/001	*C.* *felis*	Hungary	Cat	Feline
CVML12/008/807/002	*C.* *felis*	Hungary	Cat	Feline
CVML12/008/807/003	*C.* *felis*	Hungary	Cat	Feline
CVML12/008/807/004	*C.* *felis*	Hungary	Cat	Feline
CVML12/008/807/005	*C.* *felis*	Hungary	Cat	Feline
CVML12/008/807/006	*C.* *felis*	Hungary	Cat	Feline
CVML12/008/807/007	*C.* *felis*	Hungary	Cat	Feline
CVML12/008/807/008	*C.* *felis*	Hungary	Cat	Feline
CVML12/008/807/009	*C.* *felis*	Hungary	Cat	Feline
CVML12/008/807/010	*C.* *felis*	Hungary	Cat	Feline
CVML12/008/807/011	*C.* *felis*	Hungary	Cat	Feline
CVML12/008/812/005	*C.* *canis*	Albania	Dog	Canine
CVML12/008/823/003	*C.* *canis*	Albania	Dog	Canine
CVML12/008/830/004	*C.* *canis*	Albania	Dog	Canine
CVML12/008/831/005	*C.* *canis*	Albania	Dog	Feline
CVML12/008/832/001	*C.* *canis*	Albania	Dog	Feline
CVML12/008/1067/002	*C.* *canis*	Hungary	Dog	Feline
CVML12/008/934/001	*C.* *canis*	Romania	Dog	Canine
CVML12/008/941/004	*C.* *canis*	Romania	Dog	Canine
CVML12/008/1193/004	*C.* *canis*	Bulgaria	Dog	Canine
CVML12/008/1196/001	*C.* *canis*	Bulgaria	Dog	Canine
CVML12/008/1196/010	*C.* *canis*	Bulgaria	Dog	Canine
CVML12/008/1196/014	*C.* *canis*	Bulgaria	Dog	Canine
CVML12/008/1197/006	*C.* *canis*	Bulgaria	Dog	Canine
CVML12/008/1198/010	*C.* *canis*	Bulgaria	Dog	Canine
CVML12/008/997/002	*C.* *canis*	Bulgaria	Dog	Canine
CVML12/008/997/005	*C.* *canis*	Bulgaria	Dog	Canine
CVML12/008/998/003	*C.* *canis*	Bulgaria	Dog	Canine
CVML12/008/998/006	*C.* *canis*	Bulgaria	Dog	Canine
CVML12/008/999/001	*C.* *canis*	Bulgaria	Dog	Canine
CVML12/008/999/002	*C.* *canis*	Bulgaria	Dog	Canine
CVML12/008/999/003	*C.* *canis*	Bulgaria	Dog	Canine
CVML12/008/999/005	*C.* *canis*	Bulgaria	Dog	Canine
CVML12/008/999/008	*C.* *canis*	Bulgaria	Dog	Canine
CVML12/008/999/022	*C.* *canis*	Bulgaria	Dog	Canine
CVML12/008/999/026	*C.* *canis*	Bulgaria	Dog	Canine
CVML12/008/999/027	*C.* *canis*	Bulgaria	Dog	Canine
CVML12/008/999/028	*C.* *canis*	Bulgaria	Dog	Canine
CVML12/008/999/029	*C.* *canis*	Bulgaria	Dog	Canine
CVML12/008/999/039	*C.* *canis*	Bulgaria	Dog	Canine
CVML12/008/1010/007	*C.* *canis*	Bulgaria	Dog	Canine
CVML12/008/1010/008	*C.* *canis*	Bulgaria	Dog	Canine
CVML12/008/1011/013	*C.* *canis*	Bulgaria	Dog	Canine
CVML12/008/1231/002	*C.* *canis*	Romania	Dog	Canine
CVML12/008/1231/003	*C.* *canis*	Romania	Dog	Canine
CVML12/008/1231/004	*C.* *canis*	Romania	Dog	Canine
CVML12/008/1234/001	*C.* *canis*	Romania	Dog	Canine
CVML12/008/1235/001	*C.* *canis*	Romania	Dog	Canine
CVML12/008/1239/002	*C.* *canis*	Romania	Dog	Canine
CVML12/008/1253/006	*C.* *canis*	Romania	Dog	Canine
CVML12/008/1254/001	*C.* *canis*	Romania	Dog	Canine
CVML12/008/1028/001	*C.* *felis*	France	Dog	Canine
CVML12/008/1028/002	*C.* *felis*	France	Dog	Canine
CVML12/008/1028/005	*C.* *felis*	France	Dog	Canine
CVML12/008/1029/001	*C.* *felis*	France	Dog	Canine
CVML12/008/1029/002	*C.* *felis*	France	Dog	Canine
CVML12/008/1029/005	*C.* *felis*	France	Dog	Canine
CVML12/008/1030/003	*C.* *felis*	France	Dog	Canine
CVML12/008/1259/001	*C.* *canis*	Romania	Dog	Canine
CVML12/008/1259/003	*C.* *canis*	Romania	Dog	Canine
CVML12/008/1302/010	*C.* *canis*	Bulgaria	Dog	Canine
CVML12/008/1302/014	*C.* *canis*	Bulgaria	Dog	Canine
CVML12/008/1302/017	*C.* *canis*	Bulgaria	Dog	Canine
CVML12/008/1314/005	*C.* *canis*	Bulgaria	Dog	Canine
CVML12/008/1314/007	*C.* *canis*	Bulgaria	Dog	Canine
CVML12/008/1314/018	*C.* *canis*	Bulgaria	Dog	Canine
CVML12/008/1314/019	*C.* *canis*	Bulgaria	Dog	Canine
CVML12/008/1314/022	*C.* *canis*	Bulgaria	Dog	Canine
CVML12/008/1314/029	*C.* *canis*	Bulgaria	Dog	Canine
CVML12/008/1314/034	*C.* *canis*	Bulgaria	Dog	Canine
CVML12/008/1314/037	*C.* *canis*	Bulgaria	Dog	Canine
CVML12/008/1314/045	*C.* *canis*	Bulgaria	Dog	Canine
CVML12/008/1314/052	*C.* *canis*	Bulgaria	Dog	Canine
CVML12/008/1317/002	*C.* *canis*	Bulgaria	Dog	Canine
CVML12/008/1324/021	*C.* *canis*	Albania	Dog	Canine
CVML12/008/1328/001	*C.* *felis*	France	Dog	Canine
CVML12/008/1333/001	*C.* *felis*	France	Dog	Canine
CVML12/008/1347/003	*C.* *canis*	Bulgaria	Dog	Canine
CVML12/008/1348/001	*C.* *canis*	Bulgaria	Dog	Canine
CVML12/008/1348/002	*C.* *canis*	Bulgaria	Dog	Canine
CVML12/008/1349/001	*C.* *canis*	Bulgaria	Dog	Canine
CVML12/008/1380/003	*C.* *canis*	Romania	Dog	Canine
CVML12/008/1383/003	*C.* *canis*	Bulgaria	Dog	Canine
CVML12/008/1383/005	*C.* *canis*	Bulgaria	Dog	Canine
CVML12/008/1383/007	*C.* *canis*	Bulgaria	Dog	Canine
CVML12/008/1383/010	*C.* *canis*	Bulgaria	Dog	Canine
CVML12/008/1383/015	*C.* *canis*	Bulgaria	Dog	Canine
CVML12/008/1383/018	*C.* *canis*	Bulgaria	Dog	Canine
CVML12/008/1383/020	*C.* *canis*	Bulgaria	Dog	Canine
CVML12/008/1384/002	*C.* *canis*	Bulgaria	Dog	Canine
CVML12/008/1384/003	*C.* *canis*	Bulgaria	Dog	Canine
CVML12/008/1385/001	*C.* *canis*	Bulgaria	Dog	Canine
CVML12/008/1385/003	*C.* *canis*	Bulgaria	Dog	Canine
CVML12/008/1386/003	*C.* *canis*	Bulgaria	Dog	Canine
CVML12/008/1386/011	*C.* *canis*	Bulgaria	Dog	Canine
CVML12/008/1386/015	*C.* *canis*	Bulgaria	Dog	Canine
CVML12/008/1386/017	*C.* *canis*	Bulgaria	Dog	Canine
CVML12/008/1386/020	*C.* *canis*	Bulgaria	Dog	Canine
CVML12/008/1386/022	*C.* *canis*	Bulgaria	Dog	Canine
CVML12/008/1386/026	*C.* *canis*	Bulgaria	Dog	Canine
CVML12/008/1386/027	*C.* *canis*	Bulgaria	Dog	Canine
CVML12/008/1386/032	*C.* *canis*	Bulgaria	Dog	Canine
CVML12/008/1386/040	*C.* *canis*	Bulgaria	Dog	Canine
CVML12/008/1386/045	*C.* *canis*	Bulgaria	Dog	Canine
CVML12/008/1386/048	*C.* *canis*	Bulgaria	Dog	Canine
CVML12/008/1386/050	*C.* *canis*	Bulgaria	Dog	Canine
CVML12/008/1394/014	*C.* *canis*	Bulgaria	Dog	Canine
CVML12/008/1466/001	*P.* *irritans*	Europe	Dog	Canine
CVML12/008/1541/007	*P.* *irritans*	Europe	Dog	Canine
CVML12/008/1542/001	*P.* *irritans*	Europe	Dog	Canine
CVML12/008/1542/003	*P.* *irritans*	Europe	Dog	Canine
CVML12/008/1542/006	*P.* *irritans*	Europe	Dog	Canine
CVML12/008/1542/011	*P.* *irritans*	Europe	Dog	Canine
CVML12/008/1542/012	*P.* *irritans*	Europe	Dog	Canine
CVML12/008/1542/013	*P.* *irritans*	Europe	Dog	Canine
CVML12/008/1542/014	*P.* *irritans*	Europe	Dog	Canine
CVML12/008/1542/015	*P.* *irritans*	Europe	Dog	Canine
CVML12/008/1542/019	*P.* *irritans*	Europe	Dog	Canine
CVML12/008/1542/024	*P.* *irritans*	Europe	Dog	Canine
CVML12/008/1542/028	*P.* *irritans*	Europe	Dog	Canine
CVML12/008/1542/029	*P.* *irritans*	Europe	Dog	Canine
CVML12/008/1544/009	*P.* *irritans*	Europe	Dog	Canine
CVML12/008/1544/025	*P.* *irritans*	Europe	Dog	Canine
CVML12/008/1544/036	*P.* *irritans*	Europe	Dog	Canine
CVML12/008/1545/006	*P.* *irritans*	Europe	Dog	Canine
CVML12/008/1546/001	*P.* *irritans*	Europe	Dog	Canine
CVML12/008/1549/001	*P.* *irritans*	Europe	Dog	Canine
CVML12/008/1551/001	*P.* *irritans*	Europe	Dog	Canine
CVML12/008/1556/002	*P.* *irritans*	Europe	Dog	Canine
CVML12/008/1556/005	*P.* *irritans*	Europe	Dog	Canine

Canine = “*D. caninum* canine genotype”, Feline = “*D. caninum* feline genotype”

**Table 2:** Details on two distinct 28S rDNA sequence variants (“canine” and “feline”) defined from fleas infected by *Dipylidium caninum*, received from the United States of America.

Sample ID	Source	Geographic origin	Host	RFLP Genotype
CVML13/004/043	*C.* *felis*	United States of America	Cat	Feline
CVML13/004/044	*C.* *felis*	United States of America	Cat	Not determined
CVML13/004/045	*C.* *felis*	United States of America	Cat	Feline
CVML13/004/046	*C.* *felis*	United States of America	Cat	Feline
CVML13/004/049	*C.* *felis*	United States of America	Cat	Not determined
CVML13/004/050	*C.* *felis*	United States of America	Cat	Feline
CVML13/004/051	*C.* *felis*	United States of America	Cat	Feline
CVML13/004/052	*C.* *felis*	United States of America	Cat	Feline
CVML13/004/053	*C.* *felis*	United States of America	Cat	Feline

Canine = “*D. caninum* canine genotype”, Feline = “*D. caninum* feline genotype”

**Table 3:** Details on two distinct 28S rDNA sequence variants (“canine” and “feline”) defined from non-invasive anal swabs collected from cats and dogs infected by *Dipylidium caninum* in South Africa.

Sample ID	Source	Geographic origin	Host	“Genotype”
CV1	Swabs	South Africa	Cat	Feline
CV2	Swabs	South Africa	Cat	Feline
CV3	Swabs	South Africa	Cat	Feline
CV4	Swabs	South Africa	Cat	Feline
CV5a	Swabs	South Africa	Cat	Feline
CV5b	Swabs	South Africa	Dog	Canine
CV7	Swabs	South Africa	Dog	Not determined
CV8	Swabs	South Africa	Dog	Canine
CV9	Swabs	South Africa	Dog	Canine
CV10	Swabs	South Africa	Dog	Canine and feline
CV11	Swabs	South Africa	Dog	Canine
CV12	Swabs	South Africa	Dog	Canine
CV13	Swabs	South Africa	Dog	Canine
CV14	Swabs	South Africa	Dog	Canine
CV16	Swabs	South Africa	Dog	Canine
CV23	Swabs	South Africa	Dog	Canine
CV24	Swabs	South Africa	Dog	Canine
CV25	Swabs	South Africa	Dog	Canine
CV26	Swabs	South Africa	Dog	Canine
CV27	Swabs	South Africa	Dog	Canine
CV28	Swabs	South Africa	Dog	Canine
CV29	Swabs	South Africa	Dog	Canine
CV30	Swabs	South Africa	Dog	Canine
CV31	Swabs	South Africa	Dog	Feline*
CV32	Swabs	South Africa	Dog	Feline*
CV33	Swabs	South Africa	Dog	Not determined
CV34	Swabs	South Africa	Dog	Feline*
CV35	Swabs	South Africa	Dog	Feline*
CV36	Swabs	South Africa	Dog	Feline*
CV37	Swabs	South Africa	Dog	Not determined
CV38	Swabs	South Africa	Dog	Feline*
CV48	Swabs	South Africa	Dog	Feline*
CV52	Swabs	South Africa	Dog	Not determined
CV53	Swabs	South Africa	Dog	Canine
CV54	Swabs	South Africa	Dog	Canine
CV57	Swabs	South Africa	Dog	Canine
CV59	Swabs	South Africa	Dog	Canine
CV60	Swabs	South Africa	Dog	Canine
CV61	Swabs	South Africa	Dog	Canine
CV64	Swabs	South Africa	Dog	Canine
CV66	Swabs	South Africa	Dog	Canine
CV67	Swabs	South Africa	Dog	Canine
CV68	Swabs	South Africa	Dog	Canine
CV70	Swabs	South Africa	Dog	Canine
CV71	Swabs	South Africa	Dog	Canine
CV72	Swabs	South Africa	Dog	Canine
CV73	Swabs	South Africa	Dog	Canine
CV74	Swabs	South Africa	Dog	Not determined
CV75	Swabs	South Africa	Dog	Canine
CV77	Swabs	South Africa	Dog	Canine
CV80	Swabs	South Africa	Dog	Canine
CV81	Swabs	South Africa	Dog	Canine
CV82	Swabs	South Africa	Dog	Canine
CV83	Swabs	South Africa	Dog	Canine
CV84	Swabs	South Africa	Dog	Canine
CV85	Swabs	South Africa	Dog	Canine
CV86	Swabs	South Africa	Dog	Not determined
CV87	Swabs	South Africa	Dog	Canine
CV88	Swabs	South Africa	Dog	Canine
CV89	Swabs	South Africa	Dog	Canine and feline
CV90	Swabs	South Africa	Dog	Canine
CV91	Swabs	South Africa	Dog	Canine
CV92	Swabs	South Africa	Dog	Canine
CV93	Swabs	South Africa	Dog	Canine
CV94	Swabs	South Africa	Dog	Canine
CV95	Swabs	South Africa	Dog	Canine
CV96	Swabs	South Africa	Dog	Canine
CV97	Swabs	South Africa	Dog	Canine
CV98	Swabs	South Africa	Dog	Canine
CV99	Swabs	South Africa	Dog	Canine
CV100	Swabs	South Africa	Dog	Canine
CV102	Swabs	South Africa	Dog	Canine
CV103	Swabs	South Africa	Dog	Canine
CV104	Swabs	South Africa	Dog	Canine
CV105	Swabs	South Africa	Dog	Canine

Canine = “*D. caninum* canine genotype”, Feline = “*D. caninum* feline genotype”

* Dogs participated in an experimental infection efficacy study, and were infected with the *D. caninum* feline genotype

The present paper reports the several steps of analysis since the original finding. Firstly, the development of restriction fragment length polymorphism (RFLP) analysis and hydrolysis probe-based genotyping assays, for genotyping *D.* *caninum* DNA. Secondly, a sensitive, non-invasive nucleic acid detection assay applied for the detection of *D.* *caninum* DNA in faeces. And finally, the complete sequencing of the mt genome from a feline host to compare the “*D. caninum* feline genotype” and the *D.* *caninum* mt genome available in GenBank.

The objectives of this study were thus to develop the necessary assays for genotyping *D.* *caninum* DNA to discriminate between the two *D.* *caninum* genotypes. These comparisons allowed the identification and breeding of the two genotypes, in order to further evaluate their biological differences (see Beugnet et al., 2018, part 2, [4]).

## Materials and Methods

### Parasites and DNA extraction

28S rDNA PCR detection of *D.* *caninum* was performed on 6116 crude flea (*C.* *felis, C.* *canis* and *Pulex irritans*) extracts as described by Beugnet *et al* [3]. A total of 192 *D.* *caninum-*positive DNA extracts were included in the present genotype analysis. A total of 57 *D.* *caninum-*positive samples were subjected to DNA sequencing. Subsequently, all positive samples (Table 1) were subjected to RFLP (restriction fragment length polymorphism) analysis.

In addition, a total of 55 fleas collected from cats were obtained from Mike Lappin (Colorado State University, USA) and 9 of the PCR positive samples were subjected to sequencing (Table 2). Adult fleas (n = 100) were also obtained from New Zealand and 2 of the PCR positive samples were subjected to sequencing. All adult fleas were supplied in 70% (v/v) ethanol.

Five adult *D.* *caninum* tapeworms were also obtained from various sources, including 2 worms from Clinvet International (South Africa), 1 worm from École Nationale Vétérinaire de Maisons-Alfort (France), and 2 worms from Guangxi University Animal Hospital (China). All adult worms were supplied in 70% (v/v) ethanol. Genomic DNA was isolated from the adult worm proglottids using the GeneJet Genomic DNA isolation kit (Thermo Fisher Scientific), according to the manufacturer’s recommendation for tissue samples.

The original *D.* *caninum* strain maintained at Clinvet International research centre on cats and fleas was included. A new *Dipylidium* sp. strain originating from proglottids collected on dogs in the village near the research centre was also included, genotyped, and maintained on dogs. The two identified genotypes are thus maintained on dogs, cats, and their fleas, respectively, at Clinvet Research Centre, allowing further studies (Table 3).

### DNA sequencing, RFLP, and hydrolysis probe genotyping of *D.* *caninum*

Primer pairs DC28S-1F and DC28S-1R [3] were used to amplify part of the 28S rDNA region from the genome of *D.* *caninum*. PCR products were subjected to Sanger sequencing and sequence assembly of both strands were performed using Geneious assembler (Geneious 8.0.5). All PCR products obtained from Beugnet et al. [3] (Table 1) were subjected to RFLP analysis. PCR product (2 μl) was subjected to direct digestion using 10 units *Stu*I restriction enzyme (New England Biolabs) in a final volume of 20 μl for 1 hour at 37°C. The complete digestion mixture was electrophoretically separated using a 2% (m/v) TAE agarose gel at 6 V/cm for 1 hour. All appropriate controls were included.

The hydrolysis probe qPCR assays to discriminate between the two genotypes were based on a qPCR assay with the following setup. Primers DC28S-1F and DC28S-1R [3] were added to a final concentration of 900 nM each. Probes D_caninum dog (FAM-GTGTGTGCACAGTC-NFQ-MGB) and D_caninum cat (VIC-CCTGTGTGTACAGTCG-NFQ-MGB) were added to a final concentration of 200 nm each. qPCR was performed using a QuantStudio 6 instrument fitted with a 384-well block in a final reaction volume of 10 μl using SsoAdvanced™ Universal Probes Supermix (Bio-Rad) under the following cycling conditions: initial denaturation of 95°C for 10 min, followed by 40 cycles of 95°C for 15 sec and 60°C for 1 min. Pre-and post-read stages at 25°C for 30 sec were included. Analysis was performed using the QuantStudio 6 Real-time PCR system Software. All appropriate controls were included.

### Non-invasive field sampling of animals: detection and genotyping of *D.* *caninum*

Ninety-nine dogs were sampled in villages under field conditions in the area surrounding Bloemfontein (Free State, Republic of South Africa). The sampling procedure entailed the gentle swabbing of the anal region (including surrounding hair) with a sterile cotton swab. Swabs were collected from dogs living with their owners and stored at room temperature until processing for DNA isolation using the GeneJet Genomic DNA isolation kit (Thermo Fisher Scientific), according to the manufacturer’s recommendation for tissue samples. PCR based detection of *D.* *caninum* DNA obtained from 99 anal swabs from dogs resulted in successful amplification of the target region.

During the study, the anal area and hair surrounding it, as well as the sleeping area of the dog were checked for signs of proglottids. Proglottids were expelled by 12 animals at the time of swabbing. The swabs were subjected to PCR analyses, and if positive for *D.* *caninum* DNA (Table 3), the dogs were individually placed in kennels at Clinvet and screened for signs of proglottids.

### Targeted nuclear DNA amplification and sequencing

Primers WormA (5’-GCGAATGGCTCATTAAATCAG-3’) and WormB (5’- CTTGTTACGACTTTTACTTCC-3’) [11] were used to amplify the 18S ribosomal DNA region from the proglottids of feline (R166 and #1431) and canine origin (CV_ref). The complete 18S region was sequenced using a primer walking strategy. Sequence alignments were performed using the MAFFT plugin in Geneious 8.0.5. Molecular phylogenetic analyses using Bayesian inference and Neighbour-Joining were performed according to Nakao et al. [14].

Primers DC28S-3F (5’-GTGGTCAGGCCTACAGGAGTCGG-3’ based on AF023120) and Universal 28S-1R (5’-CCTGTCTCACGACGGTCTAAACCCAG-3’ based on *Hymenolepis diminuta* 28S rDNA sequence, AY157181) were used to amplify approx. 2.4 kb of the 28S rDNA region, followed by primer walking to sequence the complete region on both strands.

### Sequencing of the mitochondrial DNA

Primer pairs DcMitoUni-1F (5’-GGGCTTGTTTGAATGGTTTGACAAGATAATTTG-3’) and DcMitoUni-1R (5’-CACTTGCTGCCAAACCATTTTAGTTAAAAAACTAAG-3’) were based on the sequence *D.* *caninum* partial mt DNA sequence isolated from spotted hyena (Accession number: KF202097) (as published by East et al. [7]).

The 842 bp PCR product was amplified using total DNA isolated from R166 (obtained from the École Nationale Vétérinaire de Maisons-Alfort) and was sequenced using Sanger methodology. The above mentioned primer pairs were reverse complemented, resulting in primers pair DcMitoUni-INV-1F (5- CTTAGTTTTTTAACTAAAATGGTTTGGCAGCAAGTG-3’) and DcMitoUni-INV-1R (5’- CAAATTATCTTGTCAAACCATTCAAACAAGCCC). These primers were used at 400 nM final concentration to amplify the remainder of the mt DNA genome using LongAmp^®^ Taq DNA Polymerase (NEB) using 50 ng total DNA as template. Thermal cycling entailed 94°C for 5 min followed by 40 cycles of 94°C for 30 sec, 59°C for 30 sec, 65°C for 12 min. The thermal cycling was concluded with a final extension of 10 min at 65°C. Purified PCR product of approx. 12 500 bp was submitted to Inqaba Biotec (South Africa) for next generation sequencing making use of Illumina’s Miseq instrument and 2 × 250 bp reads using the MiSeq Reagent Kit v2. Primers F_mtDNA-F (TTCTTGAAGTTTGTCTGTCTGTTT) and F_mtDNA-R (AAGCAGCACATAGACTTAGCTT) were used to amplify 1126 bp, including the DcMitoUni-INV-1F and DcMitoUni-INV-1R primer binding sites and the PCR product was subjected to Sanger sequencing.

### Mitochondrial genome sequence analysis

Illumina Miseq and Sanger sequencing data were assembled, after quality trimming, using the Geneious assembler (Geneious 8.0.5). Contigs were mapped to the only available *D.* *caninum* mt DNA genome (AB732959), using Geneious assembler. Annotation and similarity percentages of the *D.* *caninum* R166 mt genome was performed using the Geneious Live annotate and predict function, making use of AB732959 as reference as well as submitting the final completed mt genome sequence to MITOS [2] for annotation. Geneious Live annotate and predict employs a BLAST-like algorithm that includes translation search where sequences are translated in all six frames and compared to the reference. Results from both automatic annotations were in agreement and were also manually checked.

The 12 protein-coding genes found in the sequenced and annotated mt genome of *D.* *caninum* R166 were translated using translation table 9 representing the echinoderm and flatworm mt code. The amino acid sequences were concatenated and compared to the concatenated amino acid sequences obtained from 53 mt genomes from Anoplocephalidae, Dipylidiidae, Hymenolepididae, Paruterinidae and Taeniidae using MAFFT and resulted in an 80.6% BLOSUM62 pairwise similarity after removal of all gaps in the alignment. Maximum Likelihood and Bayesian Inferences analysis were used to construct trees as described by Guo [9] using *Schistosoma japonicum* derived data as the outgroup.

The 12S rDNA sequence from the newly generated *D.* *caninum* feline genotype mt sequence was compared to *D.* *caninum* 12S rDNA sequences recently deposited and analyzed by Low et al. [13] using the Mr Bayes (HKY85 substitution model; 1 000 000 chain length and 25% burn-in length) and *Schistocephalus solidus* as the outgroup.

## Results

### Sequencing of the 28S and 18S ribosomal DNA regions.

DNA sequence analysis of a 655 bp region of the 28S ribosomal DNA region used in the *D.* *caninum* detection PCR (see Table 4) resulted in the identification of two unique groups when these DNA sequences were compared to the GenBank reference sequence (Table 5). One group exhibited 99.5% DNA sequence identity towards the published reference sequence (AF023120) and 100% DNA sequence identity towards the canine derived *D.* *caninum* isolated at Clinvet (MH040832), whereas the other group (i.e. “feline genotype”) exhibited a 93.5% sequence identity towards the published reference sequence. As described below in detail, the “100% identity group” DNA isolates were almost all of dog and dog flea origin, whereas the “93.5% identity group” DNA extracts almost all came from cats and fleas collected on cats.

**Table 4:** Percentage DNA sequence identity obtained from approximately 650 bp PCR product in *D.* *caninum* positive samples.

	*D.* *caninum* AF23120	*D.* *caninum* canine genotype	*D.* *caninum* feline genotype
*D.* *caninum* *(*AF23120		99.5%	93.5%
*D.* *caninum* canine genotype	99.5%		94.1%
*D.* *caninum* feline genotype	93.5%	94.1%	

**Table 5:** Sample detail table providing GenBank accession numbers.

Sample ID	Source	Geographic origin	Host	Sequence target	GenBank accession number
CVML12_008_023	*C.* *felis*	Slovenia	Cat	650 bp 28S rDNA	MH040824
CVML12_008_034	*C.* *felis*	Slovenia	Cat	650 bp 28S rDNA	MH040825
CVML12_008_041	*C.* *felis*	Slovenia	Cat	650 bp 28S rDNA	MH040826
CVML12_008_118	*C.* *felis*	France	Cat	650 bp 28S rDNA	MH040827
CVML12_008_193	*C.* *felis*	France	Dog	650 bp 28S rDNA	MH040828
CVML12_008_265	*C.* *felis*	Hungary	Cat	650 bp 28S rDNA	MH040829
CVML12_008_277	*C.* *felis*	Germany	Cat	650 bp 28S rDNA	MH040830
CVML12_008_279	*C.* *felis*	Portugal	Cat	650 bp 28S rDNA	MH040831
CVML12_008_CV_dog	Worm	South Africa	Dog	650 bp 28S rDNA	MH040832
CVML12_008_006	*C.* *felis*	Czech Republic	Cat	650 bp 28S rDNA	MH040833
CVML12_008_198	*C.* *felis*	France	Dog	650 bp 28S rDNA	MH040834
CVML12_008_231	*C.* *felis*	France	Dog	650 bp 28S rDNA	MH040835
CVML12_008_364	*C.* *felis*	Sicily	Dog	650 bp 28S rDNA	MH040836
CVML12_008_365	*C.* *felis*	Sicily	Dog	650 bp 28S rDNA	MH040837
CVML12_008_642	*C.* *felis*	Sicily	Dog	650 bp 28S rDNA	MH040838
CVML12_008_366	*C.* *felis*	Sicily	Dog	650 bp 28S rDNA	MH040839
CVML12_008_397	*C.* *felis*	Sicily	Dog	650 bp 28S rDNA	MH040840
CVML12_008_474	*C.* *felis*	France	Dog	650 bp 28S rDNA	MH040841
CVML12_008_523	*C.* *felis*	Sicily	Dog	650 bp 28S rDNA	MH040842
CVML12_008_530	*C.* *felis*	Sicily	Dog	650 bp 28S rDNA	MH040843
CVML12_008_601	*C.* *felis*	Sicily	Dog	650 bp 28S rDNA	MH040844
CVML12_008_373	*C.* *felis*	Sicily	Dog	650 bp 28S rDNA	MH040845
CVML12_008_539	*C.* *felis*	Sicily	Dog	650 bp 28S rDNA	MH040846
CVML12_008_550	*C.* *felis*	Sicily	Dog	650 bp 28S rDNA	MH040847
CVML12_008_598	*C.* *felis*	Sicily	Dog	650 bp 28S rDNA	MH040848
CVML12_008_603	*C.* *felis*	Sicily	Dog	650 bp 28S rDNA	MH040849
CVML12_008_632	*C.* *felis*	Sicily	Dog	650 bp 28S rDNA	MH040850
CVML12_008_246	*C.* *felis*	France	Dog	650 bp 28S rDNA	MH040851
CVML12_008_105	*C.* *felis*	France	Cat	650 bp 28S rDNA	MH040852
CVML13_004_043	*C.* *felis*	USA	Cat	650 bp 28S rDNA	MH040853
CVML13_004_044	*C.* *felis*	USA	Cat	650 bp 28S rDNA	MH040854
CVML13_004_045	*C.* *felis*	USA	Cat	650 bp 28S rDNA	MH040855
CVML13_004_046	*C.* *felis*	USA	Cat	650 bp 28S rDNA	MH040856
CVML13_004_049	*C.* *felis*	USA	Cat	650 bp 28S rDNA	MH040857
CVML13_004_050	*C.* *felis*	USA	Cat	650 bp 28S rDNA	MH040858
CVML13_004_051	*C.* *felis*	USA	Cat	650 bp 28S rDNA	MH040859
CVML13_004_052	*C.* *felis*	USA	Cat	650 bp 28S rDNA	MH040860
CVML13_004_053	*C.* *felis*	USA	Cat	650 bp 28S rDNA	MH040861
CVML12_SW_1	Swab	South Africa	Dog	650 bp 28S rDNA	MH045463
CVML12_SW_2	Swab	South Africa	Dog	650 bp 28S rDNA	MH045464
CVML12_SW_3	Swab	South Africa	Dog	650 bp 28S rDNA	MH045465
CVML12_SW_4	Swab	South Africa	Dog	650 bp 28S rDNA	MH045466
CVML12_SW_5	Swab	South Africa	Dog	650 bp 28S rDNA	MH045467
CVML12_SW_6	Swab	South Africa	Dog	650 bp 28S rDNA	MH045468
CVML12_SW_7	Swab	South Africa	Dog	650 bp 28S rDNA	MH045469
CVML12_SW_8	Swab	South Africa	Dog	650 bp 28S rDNA	MH045470
CVML12_SW_9	Swab	South Africa	Dog	650 bp 28S rDNA	MH045471
CVML14_072_004	*C.* *felis*	New Zealand	Cat	650 bp 28S rDNA	MH045472
CVML14_072_005	*C.* *felis*	New Zealand	Cat	650 bp 28S rDNA	MH045473
CVML12_008_1424	Worm	France	Cat	650 bp 28S rDNA	MH045474
CVML12_008_1430	Worm	China	Cat	650 bp 28S rDNA	MH045475
CVML12_008_1431	Worm	China	Cat	650 bp 28S rDNA	MH045476
CVML12_008_cat_CV1	Worm	South Africa	Cat	650 bp 28S rDNA	MH045477
CVML12_008_cat_CV2	Worm	South Africa	Cat	650 bp 28S rDNA	MH045478
CVML12_008_cat_CV3	Worm	South Africa	Cat	650 bp 28S rDNA	MH045479
CVML12_008_cat_CV4	Worm	South Africa	Cat	650 bp 28S rDNA	MH045480
CVML12_008_cat_CV5a	Worm	South Africa	Cat	650 bp 28S rDNA	MH045481
CVML12_008_1424_R166	Worm	France	Cat	2.4 kb 28S rDNA	MH045482
CVML12_008_1431	Worm	China	Cat	2.4 kb 28S rDNA	MH045483
CVML12_008_CV_dog	Worm	South Africa	Dog	2.4 kb 28S rDNA	MH045484
CVML12_008_1424_R166	Worm	France	Cat	2.4 kb 18S rDNA	MG582181
CVML12_008_1431	Worm	China	Cat	2.4 kb 18S rDNA	MG582183
CVML12_008_CV_dog	Worm	South Africa	Dog	2.4 kb 18S rDNA	MG582184
CVML12_008_1424_R166	Worm	France	Cat	Mitochondrial genome	MG587892

No intra-group differences in DNA sequencing could be observed for any of the samples analyzed using the 655 bp PCR product. Sequence submission to GenBank was under accession numbers indicated in Table 5.

The new “93.5% identity group” corresponds to the feline associated *D.* *caninum* samples from the USA, New Zealand, Europe, China and South Africa [with no intrinsic variation related to geographical origin (see Discriminant RFLP analysis below)]. For the clarity of the analysis, we propose to name it “*D.* *caninum* feline genotype”.

The “100% identity group” exhibited no intrinsic variation related to geographical origin between all samples from Europe and South Africa. It corresponds to the DNA extracts from infected dogs or fleas collected on dogs (see discriminant RFLP analysis below); therefore we propose to name it “*D.* *caninum* canine genotype”.

Amplification and sequence analysis of approx. 2.4 kb 28S rDNA region were performed for two of the “*D.* *caninum* feline genotype” representatives and no sequence difference was detected with the 3 ambiguous nucleotides identical between two feline genotypes. The *D.* *caninum* canine genotype yielded a sequence with 28 ambiguous nucleotides detected. In spite of the ambiguous nucleotides, a non-ambiguous DNA sequence comparison resulted in differences of 2% (with a 3.1% difference when ambiguous nucleotides were included) between the “*D.* *caninum* feline genotypes” (MH045482; MH045483) and the “*D.* *caninum* canine genotype” (MH045484) for the 2437 bp compared, with a 12 bp and a 4 bp insertion/deletion event detected.

**Figure 1:**
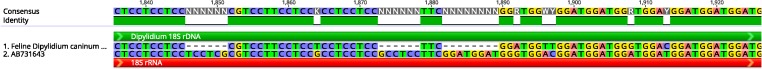
Conserved insertion/deletion events (indicated by −) present in the 18S rDNA feline genotype representing feline associated *D.* *caninum* (bottom sequence) when compared to the canine genotype (second sequence from the bottom) representing the canine associated *D.* *caninum*.

Amplification of the 18S rDNA region resulted in the amplification of 2.4 kb DNA fragments. 18S rDNA sequence analysis performed on two “*D.* *caninum* feline genotype” (MG582181, MG582183) representatives revealed 2.7% differences with the canine genotype (MG582184) with 2 conserved 6 bp and one 8 bp insertions/deletions detected in the feline sequence when compared to the canine sequence (Figure 1). Pairwise sequence comparison also indicate that the *D.* *caninum* canine and feline genotypes share sequence identity to a level that can be observed for different species of cestodes when comparing 18S rDNA sequences (Table 6). Phylogenetic analysis based on the 18S rDNA sequences were conducted using sample sets from previous studies [14] and analysis of the sequence identity and patristic distance between the feline genotype, the canine genotype and other cestodes indicated that the sequence identity and patristic distance between the *D.* *caninum* feline genotypes and the *D.* *caninum* canine genotype was larger than the patristic distance observed between *Taenia serialis* and *Taenia modoquae*; *Taenia saginata* and *Taenia asiatica*; *Taenia martis* and *Taenia twitchelli* as well as between *Echinococcus canadensis* and *Echinococcus ortleppi* (Table 6). This suggests that the two *D.* *caninum* genotypes are genetically (based on the 18S rDNA sequence data) more distant from a common ancestor than the different pairs of cestode species mentioned above.

**Table 6:** 18S rDNA sequence identity and patristic distance observed between the *D.* *caninum* feline and canine genotypes and different cestodes.

Organisms compared	Accession numbers	Pairwise DNA sequence identity	Patristic distance
*D.* *caninum* feline genotype: *D.* *caninum* canine genotype	MG582181: MG582184	97.3	0.024
*T.* *serialis: T.* *modoquae*	AB731620: AB731623	97.6	0.021
*T.* *saginata: T.* *asiatica*	AB731616: AB731617	97.8	0.011
*T.* *martis: T.* *twichelli*	AB731625: AB731626	96.6	0.021
*E.* *canadensis: E.* *ortleppi*	AB731642: AB731641	99.0	0.013

### RFLP and hydrolysis probe genotyping assays.

Sequence data from 28S rDNA region targeted for the PCR revealed the presence of conserved nucleotide differences when comparing the two genotypes. These conserved differences allowed the design of an RFLP assay using the restriction enzyme *Stu*I to generate a product that would allow direct discrimination between the two groups. The *Stu*I recognition occurs twice in the feline genotype and will result in the fragmentation of the 653 bp feline PCR product into 404 bp, 223 bp and 26 bp, and occurs only once in the reference canine genotype, yielding 629 bp and 26 bp fragments from the 655 bp PCR canine product, thereby allowing a clear discrimination between the two identified groups. RFLP analysis was also performed on the 57 sequence verified samples to validate the RFLP assay for accuracy. All results were in concordance.

*D.* *caninum* PCR positive samples (n = 192) from the previous study [3] (Table 1) were subjected to this discriminant RFLP analysis. Results indicated that 98.4% of *D.* *caninum* infected *C.* *felis* fleas collected from dogs were genotyped as the canine genotype, while only 1.6% of the positive fleas were genotyped as belonging to the feline genotype. 100% of infected *Pulex irritans* and *C.* *canis* fleas were harbouring the canine genotype of *D.* *caninum*.

RFLP analysis also showed that 90.7% of infected fleas from cats were genotyped as belonging to the feline genotype with only 9.3% of *D.* *caninum* infected fleas from cats exhibiting the canine genotype.

A hydrolysis probe genotyping assay, based on the validated PCR screening assay, was developed to allow for high throughput genotyping of the two identified groups. A 2 bp insertion/deletion present in the 653/655 bp PCR target region served as a target site for the genotyping probes. The hydrolysis probe genotyping assay was validated on samples that were subjected to Sanger sequencing and RFLP analyses. All three assays were 100% in agreement. The hydrolysis probe genotyping assays were also able to discriminate between homozygous for the “canine genotype”, homozygous for the “feline genotype” and an artificial heterozygous mix of DNA from both genotypes. This technique could serve to identify the possibility or not of hybridization between the two genotypes in definitive hosts, as cats and dogs are often present together in a same household [4].

### Identification and isolation of a *Dipylidium* canine genotype strain at Clinvet International

In order to collect a local canine genotype of *D.* *caninum* and to assess the possibility of using the PCR technique on anal swabs, 38 flea-infested dogs living in the village near ClinVet were assessed through a veterinary consultation (Table 3). Twelve dogs expelling *Dipylidium* proglottids were placed in kennels at ClinVet. Anal swabbing was conducted on all of these dogs and the swabs were subjected to PCR analyses. All 12 animals that were diagnosed infected in the field were also diagnosed positive by PCR on swabs. The 26 other dogs classified as not expelling proglottids during field direct examination, were found positive by swab PCR. These 26 dogs started to expel proglottids later in the next few days. The proglottids were genotyped to confirm the canine genotype, and were then pooled to start breeding the canine genotype colony at Clinvet by passing through fleas and dogs.

### Mitochondrial DNA amplification of sequence analysis.

Total DNA extract from adult *D.* *caninum* R166 isolate (representing the feline genotype based on 18S and 28S sequence analysis and genotyping) was used to amplify the complete mt genome in two fragments. The partial feline genotype 842 bp fragment revealed a 99% identity towards KF202097, the partial sequence obtained from the spotted hyena [7], and only an 89% identity towards the completed *D.* *caninum* mt genome from the reference group, i.e. NC_021145; “canine genotype”.

**Figure 2:**
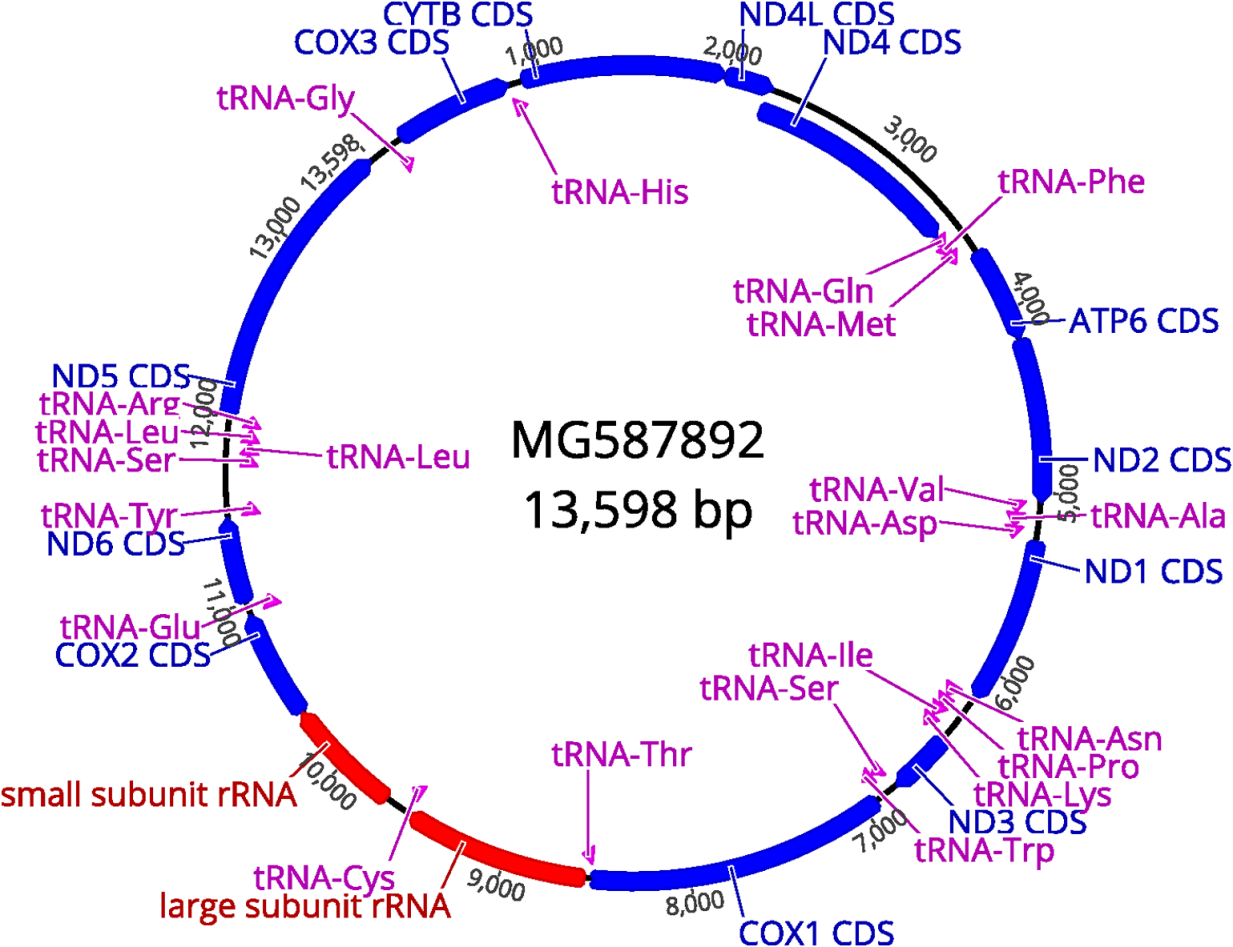
Graphical representation of the complete mitochondrial genome of *D.* *caninum* R166 (MG587892) including the organization and direction of 36 genes within the mitochondrial genome.

The remainder of the mt genome was amplified by simply reverse complementing both primers used to amplify the 842 bp fragment. The approx. 13 kb fragment was sequenced using Illumina chemistry resulting in 215 263 reads with a mean read length of 196 bp resulting in > 3000X coverage of the expected mt genome. Mapping the reads to the *D.* *caninum* mt genome (AB732959) resulted in assembly of the complete mt genome from *D.* *caninum* R166 (representing the “cat genotype”) with an mt genome size of 13 598 bp (Figure 2; GenBank accession number: MG587892). Nucleotide analysis revealed an overall base composition of 22.3% A, 8.8% C, 19.7% G and 49.1% T, resulting in a low GC content of 28.5%.

Direct DNA comparison of the complete mtDNA genomes of *D.* *caninum* R166 (“feline genotype” MG587892) and the reference *D.* *caninum* mtDNA genome (AB732959 and NC_021145; “canine genotype”) indicate only a 78.7% identity on the DNA level. The DNA sequences of AB732959 and NC_021145 are identical, but they are annotated differently.

Analyses of the CDS, rRNA and tRNA regions (36 in total) with the specific associated identities between the two mt genomes are represented in Table 7. The ATP8 coding gene, present in mammalian mt genomes, could not be observed in the *D.* *caninum* mt genome, which is in agreement with published tapeworm mt genomes.

**Table 7:** Results following analysis of the CDS, rRNA and tRNA regions (36 in total) with the specific associated identities between the two mitochondrial genomes.

Name	Type	Start (codon)	Stop (codon)	Length	DNA Identity
atp6	CDS	3658 (ATG)	4173 (TAA)	516	76.94%
Cob	CDS	899 (ATG)	1993 (TAA)	1095	81.70%
cox1	CDS	6926 (ATG)	> 8527 (*)	> 1602	88.30%
cox2	CDS	10357 (ATG)	10932 (TAA)	576	88.19%
cox3	CDS	182 (ATG)	828 (TAA)	657	80.53%
nad1	CDS	5267 (ATG)	6160 (TAA)	894	84.79%
nad2	CDS	4183 (ATG)	5054 (TAA)	872	84.52%
nad3	CDS	6445 (ATG)	6789 (TAA)	345	81.40%
nad4	CDS	2220 (ATG)	3467 (TAG)	1248	78.30%
nad4L	CDS	1993 (ATG)	2253 (TAG)	261	84.29%
nad5	CDS	12021 (ATG)	13586 (TAG)	1566	76.40%
nad6	CDS	11010 (ATG)	11465 (TAA)	456	79.17%
rrnL rRNA	rRNA	8563	9559	997	87.80%
rrnS rRNA	rRNA	9702	10343	642	87.30%
trnA	tRNA	5123	5193	71	81.69%
trnC tRNA	tRNA	9560	9623	64	75.00%
trnD tRNA	tRNA	5200	5265	66	87.88%
trnE tRNA	tRNA	10938	11004	67	75.00%
trnF tRNA	tRNA	3529	3589	61	86.15%
trnG tRNA	tRNA	117	179	63	87.69%
trnH tRNA	tRNA	829	894	66	89.71%
trnI tRNA	tRNA	6300	6362	63	92.19%
trnK tRNA	tRNA	6376	6440	65	84.62%
trnL (CUN) tRNA	tRNA	11804	11865	62	84.62%
trnL (UUR) tRNA	tRNA	11874	11937	64	83.08%
trnM tRNA	tRNA	3586	3653	68	90.00%
trnN tRNA	tRNA	6169	6232	64	91.04%
trnP tRNA	tRNA	6239	6300	62	89.23%
trnQ tRNA	tRNA	3470	3538	69	93.65%
trnR tRNA	tRNA	11956	12015	60	80.00%
trnS (AGN) tRNA	tRNA	6798	6856	59	81.67%
trnS (UCN) tRNA	tRNA	11740	11799	60	90.32%
trnT tRNA	tRNA	8528	8589	62	87.30%
trnV tRNA	tRNA	5055	5117	63	92.42%
trnW tRNA	tRNA	6860	6920	61	87.30%
trnY tRNA	tRNA	11473	11538	66	89.39%

* stop codon not determined.

No STOP codon could be detected for the COX1 protein-coding region from MG587892 and differently annotated COX1 encoding sequences are reported for AB732959 and NC_021145. Both COX1 annotations for the “canine genotype” mt DNA are under review from GenBank (GenBank email communication).

### Mitochondrial phylogenetic analysis.

**Figure 3:**
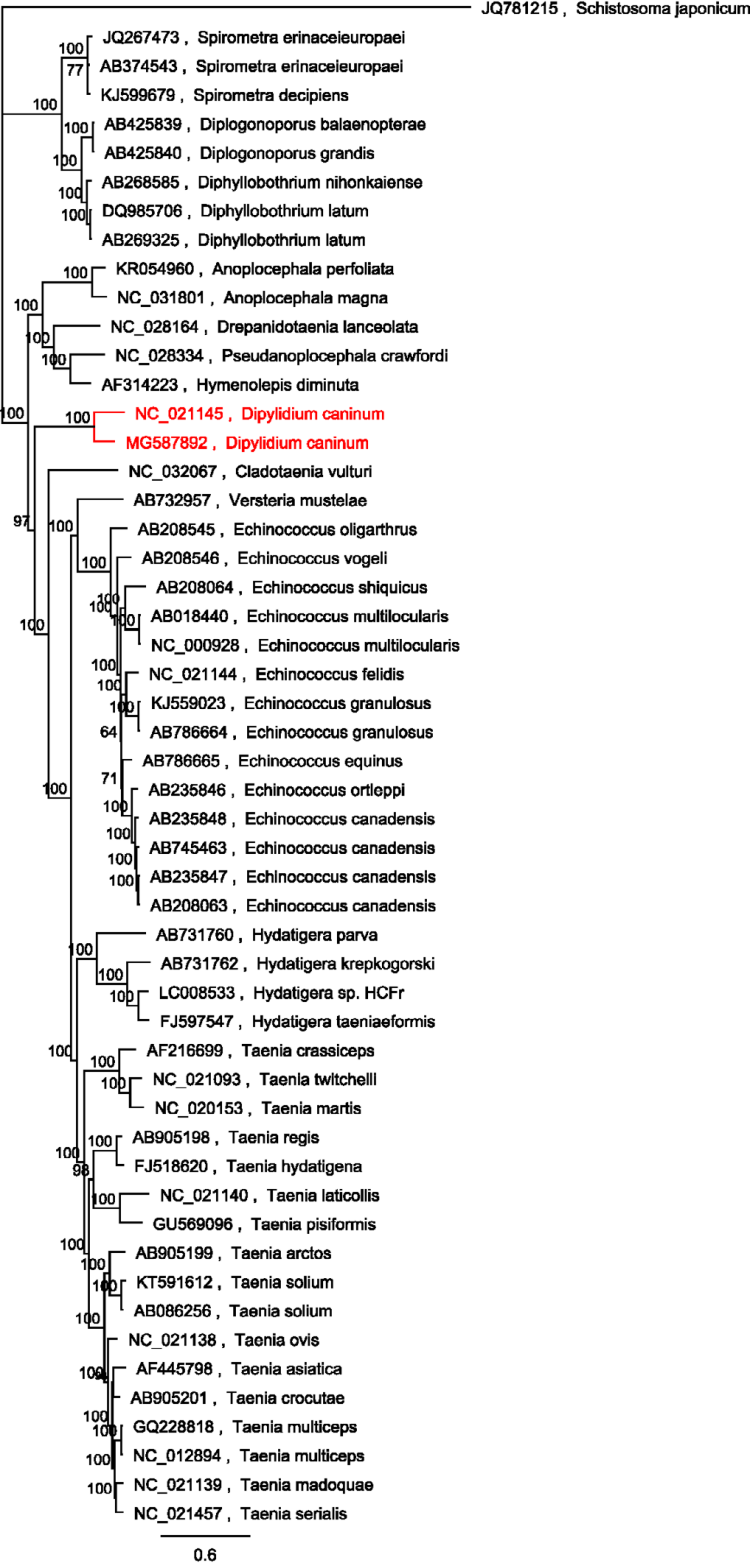
Tree obtained after concatenation of the 12 protein-coding genes from *D.* *caninum* R166 and after those of 52 other tapeworms were subjected to multiple alignment, followed by maximum likelihood and Bayesian inference analysis (average standard deviation of split frequencies was below 0.005), using *Schistosoma japonicum* as the outgroup.

Mitochondrial genomes used by Guo [9], including any updated and additional genomic DNA sequences, were downloaded from GenBank and used in all subsequent analyses. Concatenation of the 12 protein-coding genes from *D.* *caninum* feline genotype (R166 adult tapeworm isolate), canine genotype, and those of 52 other tapeworms were subjected to multiple alignment followed by maximum likelihood and Bayesian inference analysis (average standard deviation of split frequencies was below 0.005) using *Schistosoma japonicum* as the outgroup. Both analysis methods exhibited the same topology and confidence and the tree obtained from the Bayesian inference is shown in Figure 3.

Protein identity analysis (based on the concatenated mt protein sequences) between the “canine genotype” and the “feline genotype” of *D.* *caninum* mt genomes revealed only an 81.8% identity between the two genotypes, which is 17.2% lower than the average protein identity calculated for the other tapeworm genotypes available from GenBank. The patristic distance of 0.33 between the two genotypes is more than 7-fold the average patristic distance of the other sequence genotypes used in the analysis. The patristic distance between the *D.* *caninum* canine and feline genotypes is larger than the patristic distance between any of the *Echinococcus* spp., *Spirometra* spp. and *Diphyllobothrium* spp. used in the analysis. This analysis provides genetic support that the shared identity and the patristic distance scores observed between the dog and the cat genotypes identify two different *Dipylidium* species, and not only intra-species genotypes.

## Discussion

The molecular characterization of *D.* *caninum* isolates collected from dogs, cats, and in infected fleas collected either from dogs or cats allowed the identification of two distinct genotypes that clearly differ from each other.

East et al., 2013, collected *D.* *caninum* proglottids from six spotted hyena [7]. Initial PCR amplification and sequencing of the 314 bp fragment indicated identical sequence data for the partial 12S mt rDNA region from all six proglottids. Comparison of 314 bp sequence data with two published *D.* *caninum* sequences revealed a high (99%) similarity to one sequence from Europe (accession number L49460.1) but a considerably lower similarity (89%) to one sequence from Asia (accession number AB031362.1). They selected one of the six samples and PCR amplified and sequenced 1176 bp of the 12S mt rDNA (accession number KF202097). Comparison of this sequence to a similar fragment from *D.* *caninum*, again revealed a relative low similarity (89%). Pairwise sequence comparison between the sequences of East et al., 2013 and our complete mt sequence of the *D.* *caninum* feline genotype (MG587892), revealed a 99.1% identity between the *D.* *caninum* isolated from the hyena (KF202097) and the *D.* *caninum* feline genotype (MG567892) isolated from a cat. When comparing these sequences to the mt genome of the *D.* *caninum* dog genotype, there is only an 88.5% and an 88.8% identity, respectively. This confirms that the *Dipylidium* isolate from hyena belongs to the “feline genotype”.

More recently, Low et al., 2017, collected ectoparasites on dogs and cats in Malaysia [13]. In this study, *C.* *felis* (92 specimens) and *Felicola subrostratus* (30 specimens) were collected from 20 cats, whereas *C. orientis* (26 specimens) and *Rhipicephalus sanguineus* *sensu lato* (120 specimens) were collected from 29 dogs. PCR utilizing the primers we published in 2014 [3] was performed to amplify the partial 28S rDNA gene region of *D.* *caninum*. They found 2% of cat fleas and 10% of cat lice infected by *D.* *caninum*. They indicated that the representative 28S rRNA sequence isolated from their flea and louse specimens (accession no. KY751956) demonstrated 95% sequence similarity with that of *D.* *caninum* (accession no. AF023120), and they suggested the existence of a second distinct species from the one available in GenBank. This 5% divergence of the approx. 650 bp region of the 28S rDNA is consistent with data reported in this study. PCR amplification and sequencing of the partial 12S rDNA gene region indicated that the 12S rDNA sequences (accession no. KY751955) were clustered together with those adult specimens isolated from red fox (accession no. L49460) and spotted hyena (accession no. KF202097). They found a high level of genetic distance (9.59%) and concluded on the existence of two clades, with a genetic divergence comparable with that of species pairs in their relatives from the genus *Echinococcus* (1.31–10.06%) [13].

**Figure 4:**
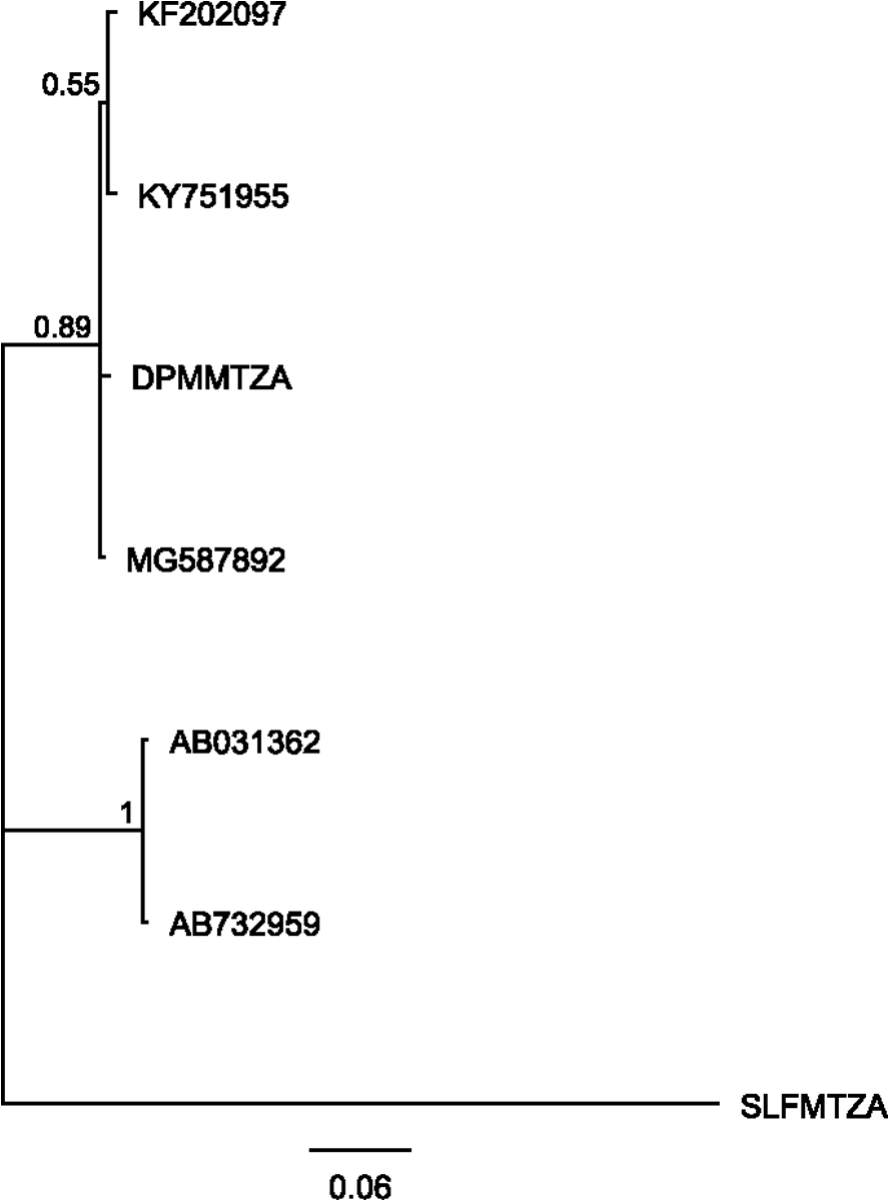
12S mt rDNA tree obtained for *D.* *caninum* sequences from GenBank using *Schistocephalus solidus* as the outgroup.

We compared the 12S mt rDNA sequence of MG587892 to *D.* *caninum* 12S mt rDNA sequences used by Low et al. [13], and Bayesian Inference phylogenetic analysis clearly clustered the *D.* *caninum* feline genotype with their *D.* *caninum* isolated from cat fleas and cat lice collected from cats (Figure 4). This clustering with the *D.* *caninum* sequence data independently obtained by Low et al. [13] confirms the clear association of the *D.* *caninum* feline genotype with cats. The hypothesis drawn by Low et al. [13] on the existence of two clades is confirmed by the presented work and corresponds to the canine and feline genotypes described in this paper.

These two genotypes are not related to geographical origin as they were found by several authors [7,13] and in the present study in *Dipylidium* sp. from all continents (i.e. North America, Europe, Asia, and Africa), Tables 1–Tables 1 to 3, but clearly to their host origin, dogs or cats (and hyena). Nevertheless, a small proportion (from 2 to 10%) of *D.* *caninum* DNA extracted from cats or *C.* *felis* fleas collected from cats, or extracted from dogs or *C.* *felis* fleas collected from dogs, belong to the other genotype. The specificity therefore does not appear to be absolute and should be studied by experimental infections in both dogs and cats (Beugnet et al., 2018, Part 2, [4]). The common presence of both cats and dogs in the same households, being infested by the same fleas (i.e. *C.* *felis*), may explain the infection of cats and dogs by both genotypes, but the different observed prevalences suggest biological adaptation. On the other hand, in *C.* *canis* and *P.* *irritans* fleas, being more specific to dogs, 100% of the infected fleas were found to harbour the canine genotype of *D.* *caninum* (Table 1).

A comparison of biological development and host preference should confirm the genetic observations (Beugnet et al., 2018, [4]). The genetic differences observed in this analysis, which show a greater distance to what is known between different species of *Taenia* or *Echinococcus*, make it possible to suggest the existence of two distinct *Dipylidium* species, which will have to be confirmed or disproved.

## Conflicts of interest

The authors declare that they have no conflicts of interest in relation to this article.
